# Cultural differences in spiritual care: findings of an Israeli oncologic questionnaire examining patient interest in spiritual care

**DOI:** 10.1186/1472-684X-13-19

**Published:** 2014-04-08

**Authors:** Michael Schultz, Doron Lulav-Grinwald, Gil Bar-Sela

**Affiliations:** 1 Division of Oncology, Rambam-Health Care Campus, Technion-Israel Institute of Technology, POB 9602, Haifa 31096, Israel; 2 Faculty of Medicine, Technion-Israel Institute of Technology, POB 9602, Haifa 31096, Israel

**Keywords:** Spiritual care, Oncology, Chaplaincy, Spiritual screening, Patient education, International, Pastoral care

## Abstract

**Background:**

As professional spiritual care (chaplaincy) is introduced to new cultures worldwide, it bears examining which elements of screening and care are universal and, for those elements showing cultural difference, to study them in each culture. No quantitative spiritual care patient study had previously been done in Israel. Our objectives were twofold: 1) to examine who wants spiritual care in Israel, including demographic and clinical variables, and to compare against other results worldwide to further develop universal screening protocols 2) to see what patients want from spiritual care specifically in the Israeli setting.

**Methods:**

Self-administered patient questionnaire examining spirituality/religiosity, interest in spiritual care (subdivided by type of care), and key demographic, social, and clinical data. The study setting was an Israeli oncology center at which spiritual care had been recently introduced.

**Results:**

Data from 364 oncology patient questionnaires found 41% interest in spiritual care, as compared to 35%-54% in American studies. Having previously been visited by a spiritual caregiver predicted patient interest in further spiritual care (AOR 2.4, 95% CI 1.2-4.6), suggesting that the new service is being well-received. Multivariate stepwise logistic regression analysis identified additional predictors of openness to receiving spiritual care: self-describing as somewhat/very spiritual vs. not spiritual (adjusted odds ratio [AOR] 3.9 and 6.3, 95% CI 1.8-8.6 and 2.6-15.1) or traditional/religious vs. secular (AOR 2.2 and 2.1, 95% CI 1.3-3.6 and 1.1-4.0); and receiving one visit a week or less from family and friends (AOR 5.6, 95% CI 2.1-15.1). These findings are in line with previous American studies, suggesting universality across cultures that could be utilized in screening. Differences in demographic data and medical condition were not significant predictors of patient interest, suggesting a cultural difference, where age and education were predictors in the American context. Levels of interest in explicitly religious or spiritual support such as prayer or addressing religious/spiritual questions were much lower than in other cultures.

**Conclusions:**

Results illustrate the demand for and satisfaction with the new Israeli service. The cross-cultural comparison found both culture-dependent and possibly universal predictors of patient interest, and found lower interest in Israel for explicitly religious/spiritual types of support.

## Background

Spiritual care addresses a key patient need [1,2] in a manner that has significant benefits, such as improved quality of life [1,3,4], well-being [5], and reduced anxiety, despair, or depression [6-8] that have been demonstrated cross-culturally [9]. Spiritual care has become an integral part of palliative care [10,11] and should be seen as an element of providing care for the whole person [12].

Professional spiritual care has been introduced to more and more countries worldwide. While spirituality is a universal phenomenon, spiritual care in a particular setting may have unique culture-specific aspects due to the specific religions and spiritual approaches to be found there. One area where we expect to find cross-cultural difference is in patients' spiritual needs. For example, in Taiwan, where the culture is heavily influenced by Taoism, Confucianism, and Buddhism, a key spiritual need expressed is facing death peacefully [13]; and in Tanzania, addressing concerns about witchcraft, devils, and curses are important spiritual needs [14,15]. Some aspects of spiritual needs are universal while others find a culture-specific expression – "spirituality is embedded within culture" [16]. For this reason, there is an ongoing effort either to develop tools that are valid for a particular cultural setting (for example, Spain, Iran, or for African-Americans [17-19]) or to establish cross-cultural validity for instruments that presumably are limited to universal needs [16,20].

The introduction of spiritual care to any new setting needs to be accompanied by research establishing the local parameters for care provision, including examining the spiritual needs of the local population. This will help address all their needs, whether they are likely to be cross-culturally universal or not. One question of particular interest in the Israeli setting is the place of religious care within spiritual care. In some countries, such as Japan [21] and Korea [9], studies of spiritual care reveal a close linking between religious care and spiritual care. But in Israel, our profession has been very concerned to distinguish between the two, following the broader definition of spirituality that has increasingly become accepted in the field in the West [11]. One qualitative study examining key Israeli stakeholders' attitudes towards spiritual care highlights the tension among Jews in Israel, "where the religious and secular publics are polarized and the secular shy away from anything that may be interpreted as religious coercion" [22]. As one nursing institution director said, " 'I don't want it to appear as if the spiritual support provider has anything to do with things such as organ donations or religion.' " Out of awareness of this concern regarding the relationship between spiritual care and religion, spiritual care in Israel has intentionally been built not on a religious framework, in contrast with some other parts of the world [23]. Our study in part examines this concern from a quantitative perspective, measuring patients' expressed spiritual needs. In another more secular society, Australia, researchers found that patients' desire to speak about their spirituality was similar to that found in the United States, a religious society, suggesting that it is important to test these assumptions [24]. One article looking at the spiritual care needs of Muslims in Israel, though not research based, suggests that this religious/secular tension is less relevant for spiritual care provision to Israeli Muslims [25].

By contrast, it is not clear to what extent appropriate screening methods need to reflect cultural differences. Numerous studies have found that patients are not receiving as much spiritual care as they would like [26], either because of a shortage of resources or because their spiritual distress remained unidentified. In the EAPC Spiritual Care Taskforce's recent large international survey of clinicians and researchers determining key research priorities, improving the means of patient screening was one of three priority areas identified [27]. There are at least two elements of screening: measuring spiritual distress and determining which patients would express a desire to receive spiritual care. The former requires demonstrating the cultural fit of the measurement tool, as has recently been done in Brazil [28], and that remains the subject of future study in Israel. The latter is one of the focal areas of the present study.

Who wants to receive spiritual care, and can those results be generalizable? In any cultural setting, researching the key demographic traits that significantly predict patient interest in spiritual care would help to streamline the process of screening. However, it bears consideration whether some of these factors are cross-cultural, and possibly do not require renewed study in each particular setting.

Several studies provide data regarding which types of patients (distinguished by demographic, cultural, sociological, or disease-related difference) are most likely to be interested in spiritual care [29-32]. Older age was a significant factor in three studies [29-31], though not in the fourth [32]. Educational level was a significant factor in every study, but it was inconsistent, with lower educational level predicting higher interest in three studies but higher educational level in the fourth [32]. Gender, marital status, ethnicity, and religion were consistently insignificant factors. Most measures of illness were insignificant predictors of patient interest, though three studies found some limited relationship [29-31]. Increased experience in receiving spiritual care predicted more positive attitudes towards spiritual care in the two studies that examined that factor [31,32]. Spirituality, religiosity, or religious practice were significant predictors in at least three of the studies [29-31]. However, all of these studies were carried out in the United States. As a result, even items that seem to be nearly always either significant or insignificant predictors of interest in spiritual care, which in theory could be integrated into a screening protocol, might not be reliably significant or insignificant in other cultural settings. This study will examine the factors predicting Israeli patient interest in spiritual care in order to compare it with the American results.

Regarding the level of interest in spiritual care, a number of American studies surveying patients in a variety of different departments found interest ranging from 35% to 54% [26,29,30,33]. However, it may not be possible to infer interest levels from a country with an established spiritual care service, like the US, to one where spiritual care is new and not well-known [22], like Israel.

There have been qualitative studies examining the challenges and accomplishments in introducing professional spiritual care in Israel over the past decade [22,23,34]. However, there have not been any quantitative studies in Israel nor studies of any kind of the question of patient interest in spiritual care prior to the present study.

Rambam is the tertiary care medical center for Haifa and its environs as well as for all of northern Israel. Haifa proper is 82% Jewish and 14% Christian, while northern Israel is 44% Jewish, 38% Muslim, 8% Druze, and 7% Christian.

## Methods

This study is a patient survey, completed independently.

### Sample and procedure

A questionnaire with 61 items to be completed independently and anonymously was distributed to patients who were in the hospital's chemotherapy or radiation outpatient treatment rooms or who were hospitalized in the Division of Oncology at Rambam Health Care Campus in Haifa, Israel. Questionnaires were not distributed in each patient area every day. On those days randomly selected for questionnaire distribution in a particular patient area, questionnaires were distributed to all patients present at the time of distribution. Distribution was done by staff not already part of the patient's care team, to limit desirability bias. Questionnaires were available in Hebrew, Arabic, Russian, and English, thereby ensuring that the vast majority of our patient population would not be excluded for reasons of language. The questionnaire was written in English and translated to Hebrew. Next it was translated to Arabic and Russian by staff in a process of translation – reverse translation to check for consistency. The study was approved by the Rambam institutional ethics review board (ref. #2999), and as per their guidelines, patient consent to self-complete the anonymous questionnaire was granted verbally.

### The questionnaire

The questionnaire contained three sections: Spiritual identity (self-defined spirituality and religiosity); Types of support provided by spiritual care, rated by importance assigned to them; and Demographic and clinical details. The cover page described spirituality and spiritual care. The full questionnaire can be viewed in the Additional file 1.

### Measures

#### Spirituality/religiosity and spiritual resources

Self-defined spirituality and religiosity were selected in two items from among the options: Not spiritual/Somewhat spiritual/Very spiritual and Secular/Traditional/Religious. The religious terminology chosen followed previous Israeli studies [35]. These three options are the standard terms used across the various religions represented, where "religious" connotes stricter observance of the religion's commandments while "traditional" connotes strong affiliation and belief without full religious observance. A fourth option from other studies, "ultra-Orthodox", was excluded since it applies to Judaism but not to the other Israeli religions.

#### Types of support the spiritual caregiver could provide

Our goal was to assess the extent to which patients valued different aspects of spiritual care. Because we were researching a new cultural setting, we wanted to ensure that we examined a substantially broad range of descriptors of spiritual care, including both "secular" and "religious" descriptors. Because the questionnaire would be completed while receiving or waiting for treatment, it needed to be shorter than existing measures. Since our goal was to establish a baseline data set for Israeli spiritual care needs and desires, we did not consider instruments designed for spiritual assessment or spiritual history taking.

This section of our questionnaire was a composite of four previously reported instruments itemizing spiritual needs that can be addressed by pastoral care. Galek et al. [36] analyzed a large cohort of studies of patient spiritual needs. Content analysis discerned seven representative constructs, from which they designed a 29-item patient survey of spiritual needs, all of which could potentially be addressed by pastoral care. Their literature review (articles from 1990–2004) was not geographically limited, but predates most of the recent efforts to verify cross-cultural validation of spiritual care instruments used [16]. Kernohan et al’s semi-structured questionnaire, based on the standards of the Association for Hospice and Palliative Care Chaplains, was conducted in an Irish hospice and asked patients about the importance for them of defined spiritual needs which the pastoral care team could help address [37]. VandeCreek’s large study [31] of 1440 patients used a shortened version of the Patient Satisfaction Instrument–Chaplaincy (PSI-C), developed in Canada, to assess patient satisfaction with various aspects of the support provided by the chaplain. Flannelly et al. [38] conducted a meta-analysis of the literature (not geographically limited) reporting key items determining patient/family satisfaction, then combined it with VandeCreek's instrument in order to create a survey measure of the effectiveness of pastoral care.

Galek, Flannelly, and Vandecreek identify, with some variation, thematic areas for the care provided by chaplains, grouped together below. We assigned the items in all four measures [31,36-38] to these thematic areas. Following the approach of Fitchett [29], we synthesized our 15-item instrument from the four instruments enumerated above [31,36-38], thereby ensuring that our questionnaire examined elements of each of these thematic areas, as follows:

##### Sensitivity/caring/support/love

Two items from Flannelly ("Listen to your concerns and show care for you"; "Show care for your family").

##### Information/decision-making/coordination

Two items from Flannelly ("Help you make difficult decisions"; "Help you obtain information or help in communicating with staff").

##### Reflecting/finding meaning

Three items from Flannelly, Kernohan, and Galek ("Help you reflect on your experience"; "Help you find meaning in your situation"; "Address spiritual or religious questions").

##### Coping/peace/hope/dignity

Five items from Flannelly, VandeCreek, and Galek ("Help you face your situation with calmness and dignity"; "Help you find hope or encouragement"; "Help you cope with your sense of loss"; "Help you cope with and adjust to the whole situation"; "Help you find strength to continue").

##### Spiritual experiential

Two items from Galek and Flannelly ("Pray with you"; "Bring a sense of spirituality into the room").

##### Activities/rituals

Appropriate to our cultural context, in which spiritual caregivers are not religious figures, in place of a question about religious rituals we added one item about common Israeli spiritual practices, such as meditation, guided imagery, music, and relaxation [39].

Patients were asked to indicate the level of importance they ascribed to each way in which the spiritual caregiver could support them. Level of importance was rated on a seven-point Likert-type scale. For our analysis, responses of 1–3 indicated the item was not important to patients, 4 was neutral, and 5–7 indicated importance.

#### Demographic and clinical details

Demographic variables collected were age, marital status, number of children, education, religion, gender, and country of birth. Sociological/behavioral characteristics assessed were level of support in living situation, level of support from family and community, level of support during hospitalization, and attendance at religious services. Clinical measures, as self-reported by respondents, were cancer diagnosis, stage of treatment, time since primary diagnosis, whether cancer had recurred, and whether cancer had metastasized. One question asked patients how worrisome their cancer is. The questions and response categories may be viewed in the article Additional file 1.

#### Attitude toward spiritual care

Respondents were asked four questions relating to their experience with and disposition toward spiritual care: 1) Have you ever had a visit from a spiritual caregiver? 2) Do you think you have a good understanding of what a spiritual caregiver is or does? 3) How important do you think it is that the oncology institute includes spiritual care in its services? 4) How open do you think you would be to a visit from the spiritual caregiver? That final question formed the primary basis for our analysis of patient interest in spiritual care.

#### Statistical analyses

Statistical analyses were performed using SPSS (Statistics Products Solutions Services) 18.0 software for Windows. Binary logistic regression was used for the calculation of the odds ratios (OR) with 95% confidence intervals (CI) and *P*-values in bivariate analysis. Multivariable stepwise logistic regression analysis was performed to assess the relationship between the patient demographic or social data and patient interest in a visit from the spiritual caregiver. The area under the receiver operating characteristic (ROC) curve was used as a measure of model discrimination. The Hosmer-Lemeshow goodness-of-fit statistic was calculated.

Comparisons between patient interest in a visit from the spiritual caregiver and interest in specific types of support were performed using the χ^2^ test. Two-tailed *P*-values of 0.05 or less were considered statistically significant.

## Results

We received 364 sufficiently complete questionnaires, a large majority of which were completed in the outpatient clinics. Questionnaires were distributed from March through August 2010 and again from April through mid-May 2011. Completion of questionnaires was voluntary. The most common reason volunteered by patients for non-completion was physical distress.

Fifty-five percent of respondents were female, and 52% were over the age of 60. Ethnic and religious orientation, as shown in Table 1, was largely in keeping with regional demographic patterns, though the Arab population may be slightly under-represented, composing 16% of respondents but approximately 25% of the regional population.

**Table 1:** Associations between patient demographic, spiritual, and clinical data and patient openness to receiving a spiritual care visit (bivariate analysis)

	**No. of patients (%)**	**No. interested in spiritual care (%)**	**Odds ratio**	**Confidence interval (95%)**	**p Value**
Gender					
Male	151 (41)	59 (39)	1.00	Ref.	
Female	186 (51)	84 (45)	1.26	0.81-1.94	0.305
Non-response	27 (7)				
Age					
Under 40	33 (9)	16 (48)	1.00	Ref.	
40-50	47 (13)	24 (51)	1.11	0.46-2.70	0.820
50-60	90 (25)	33 (37)	0.62	0.28-1.38	0.237
60-65	62 (17)	27 (44)	0.82	0.35-1.91	0.646
66-70	43 (12)	15 (35)	0.57	0.23-1.44	0.233
Over 70	79 (22)	31 (39)	0.69	0.30-1.56	0.367
Non-response	10 (3)				
Marital status					
Single	19 (5)	7 (37)	1.00	Ref.	
Married	264 (73)	108 (41)	1.17	0.45-3.06	0.752
Divorced	40 (11)	19 (48)	1.56	0.51-4.75	0.442
Widowed	33 (9)	14 (42)	1.26	0.40-4.03	0.693
Non-response	8 (2)				
Educational level					
Primary school	49 (13)	22 (45)	1.00	Ref.	
High school	115 (32)	48 (42)	0.88	0.45-1.73	0.708
More than high school	184 (51)	74 (40)	0.83	0.44-1.56	0.554
Non-response	16 (4)				
Religion					
Jewish	276 (76)	119 (43)	1.00	Ref.	
Muslim	34 (9)	16 (47)	1.19	0.58-2.43	0.633
Druze	11 (3)	3 (27)	0.50	0.13-1.93	0.317
Arab Christian	12 (3)	5 (42)	0.96	0.30-3.09	0.941
Other Christian	8 (2)	3 (38)	0.80	0.19-3.43	0.767
Other	8 (2)	2 (25)	0.45	0.09-2.25	0.328
Non-response	15 (4)				
Place of birth					
Israel	168 (46)	77 (46)	1.00	Ref.	
FSU	70 (19)	27 (39)	0.72	0.40-1.30	0.277
Europe	47 (13)	15 (32)	0.54	0.27-1.09	0.084
Middle East/N. Africa	51 (14)	22 (43)	0.87	0.45-1.67	0.677
Other	14 (4)	5 (36)	0.64	0.20-2.01	0.441
Non-response	14 (4)				
Spirituality					
Not spiritual	58 (16)	9 (16)	1.00	Ref.	
Somewhat spiritual	203 (56)	87 (43)	4.17	1.94-8.94	<0.001*
Very spiritual	76 *(*21)	46 (61)	8.35	3.58-19.47	<0.001*
Non-response	27 (7)				
Religiousness					
Secular	146 (40)	48 (33)	1.00	Ref.	
Traditional	135 (37)	63 (47)	1.79	1.10-2.90	0.019*
Religious	61 (17)	33 (54)	2.41	1.31-4.43	0.005*
Non-response	22 (6)				
Time since primary diagnosis					
<4 weeks	25 (7)	11 (44)	1.00	Ref.	
1-3 months	59 (16)	21 (36)	0.70	0.27-1.82	0.469
3-6 months	76 (21)	33 (43)	0.98	0.39-2.43	0.960
>6 months	176 (48)	79 (45)	1.04	0.45-2.41	0.934
Non-response	28 (8)				

*p <0.05.

Data not showing significant correlation not included in table: disease recurrence, metastatic disease, status of oncology treatment.

In response to the question, "How important do you think it is that the oncology institute includes spiritual care in its services?", 60% of patients felt it was important for spiritual care to be offered, regardless of their own personal interest in the service.

In response to the question, "How open do you think you would be to a visit from the spiritual caregiver?", 41% of patients were positively predisposed to such a visit (25% definitely interested; 16% possibly interested). Bivariate analysis of the other items in relation to this question determined the significant predictors of interest in spiritual care.

As shown in Table 1, none of the demographic or clinical items predicted a particular degree of openness to spiritual care. However, items describing one's own level of spirituality or religiosity were strongly significant in our bivariate analysis. Patients self-describing as "somewhat spiritual" or "very spiritual" were 4.2 and 8.4 times as likely (odds ratio [OR]) to be interested in spiritual care as those who were "not spiritual" (95% CI, 1.94-8.94 and 3.58-19.47; *P* < 0.001), while those who were "traditional" or "religious" were 1.8 and 2.4 times as likely, respectively (OR), to be interested as those who were "secular" (95% CI, 1.10-2.90 and 1.31-4.43; *P* = 0.019 and 0.005, respectively).

Certain experiential factors were significant predictors of an interest in spiritual care in our bivariate analysis, as listed in Table 2. Hospitalized patients receiving one visit a week or less from family or friends were more likely to want a spiritual care visit than those visited almost daily (OR, 3.9; 95% CI, 1.54-9.63; *P* = 0.004). Patients who had been visited previously by spiritual caregivers were more likely to want another visit, compared to those who had never experienced spiritual care (OR, 3.9; 95% CI, 2.0-7.8; *P* < 0.001). Those who felt they had a good understanding of what spiritual care is were more likely to be open to a visit than those who felt they did not understand it (OR, 2.9; 95% CI, 1.8-4.8; *P* < 0.001). The other sociological or experiential factors examined (living alone; supportive community; family nearby; attending religious services) were not significant predictors.

**Table 2:** Significant associations between experiential factors and patient openness to receiving a spiritual care visit, bivariate analysis

	**No. of patients (%)**	**Interested in spiritual care (%)**	**Odds ratio**	**Confidence interval (95%)**	**p Value**
Frequency of Visitors (if hospitalized)					
Almost every day	174 (48)	71 (41)	1.00	Ref.	
Few times a week	22 (6)	10 (45)	1.18	0.48-2.88	0.715
Once a week or less	26 (7)	19 (73)	3.85	1.54-9.63	0.004*
Not hospitalized	99 (27)	41 (41)	1.00	0.61-1.65	0.996
Non-response	43 (12)				
Previous visit from spiritual caregiver?					
No	312 (86)	115 (37)	1.00	Ref.	
Not sure	9 (2)	5 (56)	2.1	0.6-8.0	0.272
Yes	43 (12)	30 (70)	3.9	2.0-7.8	<0.001*
Understanding of what a spiritual caregiver is/does					
No	102 (28)	27 ( 26)	1.00	Ref.	
Unsure	37 (10)	12 (32)	1.4	0.6-3.2	0.418
Yes	225 (62)	113 (50)	2.9	1.8-4.8	<0.001*

*p <0.05.

We attempted to construct a model of the patient most likely to be open to receiving spiritual care, using multivariate logistic regression. Because we found co-linearity between religiosity and spirituality (*P* < 0.001), we could not use the same model for both variables. Table 3 presents two possible models side-by-side, one including religiosity and excluding spirituality, and the other including spirituality and excluding religiosity. The other predictors previously identified (frequency of friend/family visits; previous visit from spiritual caregiver; understanding of what spiritual care is) retained their statistical significance in the multivariate analysis in both models.

**Table 3:** Prediction of openness to spiritual care visit, multivariate logistic regression model

**Variable**	**Response**	**Coefficients**	**p Value**	**Adjusted OR**	**Confidence interval**	**Variable**	**Response**	**Coefficients**	**p Value**	**Adjusted OR**	**Confidence interval**
Religiosity	Secular		0.004	1.00		Spirituality	Not spiritual		<0.001	1.0	
	Traditional	0.79	0.002	2.2	1.3-3.6						
	Religious	0.74	0.024	2.1	1.1-4.0		Somewhat spiritual	1.37	0.001	3.9	1.8-8.6
							Very spiritual	1.83	<0.001	6.3	2.6-15.1
Frequency of visits	Once/wk or less	1.73	0.001	5.6	2.1-15.1	Frequency of visits	Once/wk or less	1.57	0.004	4.8	1.7-13.9
Previous visit from spiritual caregiver	Yes	0.86	0.011	2.4	1.2-4.6	Previous visit from spiritual caregiver	Yes	0.75	0.034	2.1	1.1-4.2
Understanding of spiritual care	Yes	1.04	<0.001	2.8	1.7-4.7	Understanding of spiritual care	Yes	0.81	0.002	2.3	1.3-3.8
Constant	Constant	-1.69	<0.001	0.19		Constant	Constant	-2.348	<0.001	0.10	
											

The importance attached to particular ways that spiritual care can be supportive significantly predicted a patient's interest in personally receiving spiritual care in every case – the higher the patient interest in a particular kind of support, the greater the interest in personally receiving spiritual care, as shown in Table 4. Among those interested in receiving spiritual care, "Help you face your situation with calmness and dignity" (74.8%) and "Show care for your family” (71.5%) were the types of support most often rated as important, while “Address spiritual or religious concerns” (47.7%), “Bring a sense of spirituality into the room” (45.7%) and “Pray with you” (30.5%) rated lowest. Even among those indifferent to a visit from the spiritual caregiver, over half the respondents rated as important 10 out of 15 types of support.

**Table 4:** Importance of specific types of support the spiritual caregiver can provide associated with openness to spiritual care visit, bivariate analysis

**Variable**	**% of those not open to visit (N=120)**	**% of those unsure about visit (N=93)**	**% of those interested in visit (N=151)**	**p value**
Help you face your situation with calmness and dignity	34.2	65.6	74.8	<0.001
Show care for your family	37.5	52.7	71.5	<0.001
Help you find strength to continue	33.3	67.7	70.9	<0.001
Help you find meaning in your situation	26.7	55.9	68.9	<0.001
Help you find hope or encouragement	30.8	66.7	68.9	<0.001
Help you obtain information or help in communicating with staff	34.2	50.5	68.2	<0.001
Listen to your concerns and show care for you	34.2	53.8	67.5	<0.001
Help you cope with and adjust to the whole situation	30.0	57.0	66.9	<0.001
Help you make difficult decisions	34.2	52.7	64.2	<0.001
Help you reflect on your experience	25.0	45.2	62.3	<0.001
Help you cope with your sense of loss	23.3	44.1	61.6	<0.001
Offer you supportive techniques like relaxation, meditation, music, and guided imagery	23.3	51.6	58.9	<0.001
Address spiritual or religious questions	15.8	37.6	47.7	<0.001
Bring a sense of spirituality into the room	15.8	38.7	45.7	<0.001
Pray with you	14.2	35.5	30.5	0.001

Note: "Important" defined as 5–7 on the 7-point Likert scale.

## Discussion

Despite the relatively recent introduction of professional spiritual care in Israel, the percentage of patients interested in the service (41%) or valuing its inclusion in the hospital's services (60%) indicate a significant positive disposition towards hospital-based spiritual care. Previous studies in hospitals where spiritual care was better established found a range of patient interest in spiritual care: 54% (rehabilitation) [33], 41% (internal medicine) [30], and 35% (medical/surgical) [29]. Despite the newness of the Israeli spiritual care service, levels of patient interest were within the range found in those more established settings. As can be seen in Table 4, the value given to addressing spiritual needs correlates with a desire for spiritual care, suggesting that patients already look positively at this service as a means of helping them with their spiritual needs. Patients' sense of understanding what spiritual care is was a predictor of their interest in the service that persisted in the multivariate analysis. This suggests the importance of education and awareness in determining public interest in the service – the more patients understood what the service has to offer, the greater their interest in receiving the service. That result parallels the finding that lack of knowledge and understanding are key factors in institutions not including spiritual care [22]. The results for patient interest may be expected to change as levels of awareness grow.

Even among those who expressed indifference to a spiritual care visit, between half and two-thirds of such patients found the kinds of support spiritual caregivers provide to be important to them for most items. Increased patient education might shift that indifference into openness, although it also could be the case that those patients’ needs are being met elsewhere.

The significance of previous experience in receiving spiritual care as a positive predictor of patient interest in the service supports previous findings [31,32]. The persistence of this factor even in the multivariate model suggests that it is not just that those who were previously likely to be interested in spiritual care continue to be interested. Rather, this finding supports the positive impact of care and suggests that it is being well received.

To what extent does the significance of demographic, medical, and social/experiential factors vary between Israel and other cultural settings, in predicting patient interest or satisfaction? As described in the Background, we are aware of four studies, all American, that measured this question. In the present study, as in those previous studies, gender, marital status, religion, and ethnicity were not predictive factors [29-32] (with the exception of "Other" ethnicity in [30]).

Older age was a significant predictor in 3 of 4 studies, but not in the present study. Perhaps its significance was confounded in our results by the fact that younger Israelis show increased spirituality [40], or it may provide evidence for a cross-cultural difference. Educational level, significant in every prior study though not always in the same direction, was not significant here, perhaps suggesting that it, too, can reflect cultural difference.

Our study was the only one to examine country of origin. Israel is a nation of immigrants from countries with widely differing cultural approaches in caring for illness and spirituality [39]. This item is of particular interest regarding the question of whether we should expect to find cross-cultural difference in patient interest around the world, and the fact that it was an insignificant factor in our study strengthens the above conclusion that variance between countries will not be significant.

As in most other studies, self-defined spirituality [30,32], or religiosity [29,30] were significant predictors of positive perceptions of spiritual care. It seems likely that these are fairly universal factors. Our study did not find significance in public religious practices, such as attendance at worship services; other studies also differed regarding the significance of that item [29-31]. It should be noted that our results showed co-linearity between spirituality and religiosity even though the questionnaire cover letter, viewable in the Additional file 1, distinguishes clearly between the two.

In examining the impact of the seriousness of the medical condition on patient interest in spiritual care, the results have not been uniform. Ledbetter's approach to spiritual screening assumes that the likely impact of the medical condition on the patient’s life is a major factor [41]. Some studies found significance in average disease length of stay [29] or severity of pain [30], but other medical factors including cancer diagnosis and comorbidity were found in those studies and elsewhere [32] not to be statistically significant. Our study did not find any of the medical factors, including recurrence, metastasis, and treatment stage, to be significant. However, the fact that respondents had to be physically able to answer the questions, even if at times with the help of family or staff member, excluded those who were in worse condition, perhaps masking the predictive significance of medical condition. In addition, because all respondents were diagnosed with cancer, we could not measure the differential impact of a cancer diagnosis to that of other illnesses.

Lucas' approach to pastoral care emphasizes community, with the expectation that lesser community support increases the need for spiritual care [42], and community is one of the key areas covered by Puchalski’s FICA tool for spiritual history taking [43]. Ledbetter's screening approach considers lack of social support to be a major factor determining low coping resources [42]. One study did not find a significant relationship between social support and patient requests for spiritual care [29], and some of the social support items we included were insignificant as well. However, Lucas' and Ledbetter's predictions of the significance of community were supported by our persistent finding that lower frequency of visits by friends and family was a predictor of patient interest in spiritual care. Identifying "lonely" patients as more likely candidates for spiritual care helps provide direction to departmental staff members and spiritual caregivers in determining whom to visit in the limit number of available staff hours.

What do these results suggest for the viability of cross-cultural screening protocols? The current data suggest that most demographic factors are consistently irrelevant, though age and education may be significant in certain cultures. The stage or severity of disease is of ambiguous utility for screening regardless of cultural setting. It should be noted that the persistent factors in the present study, including spirituality/religiousness and support from family/friends, largely match the factors identified in the FICA tool, which may prove to be a valuable cross-cultural measure.

In looking at spiritual needs and the kinds of spiritual care support patients most valued, there was a significant cultural difference, as predicted, regarding explicitly religious/spiritual items. There were four such items, and they were the four lowest-rated. Prayer ranked last in our study among kinds of spiritual support desired, at 30% of those interested in spiritual care, whereas in America prayer was the most common intervention expected of spiritual caregivers [44], desired by 74% of those interested in spiritual care in one study [29]. Other low-ranked items were addressing spiritual/religious questions (35% overall) and bringing a sense of spirituality to the room (46% of those interested in the service), versus 61% of Irish hospice patients [37] and 78% of religious Japanese bereaved family members [21], respectively. Only 43% of patients overall were interested in relaxation, meditation, music, or guided imagery, which could be generally characterized as spiritual techniques. Although interest in these religious/spiritual items predicted interest in spiritual care, there were many similarly predictive items, not explicitly religious/spiritual, endorsed by a much larger percentage of the population. The spiritual care desired by Israeli patients is not limited or primarily directed to the explicitly religious/spiritual realm.

The main methodological limitation of the study was the population response bias. The questionnaires were offered to all patients currently hospitalized or in the treatment clinic at that moment. However, we did not gather demographic data on those who chose not to complete the survey to compare with those who did, and did not analyze the bias in who chose to participate. We also did not record what percent of patients approached chose not to complete a questionnaire, for physical or other reasons.

The question regarding "Frequency of Visitors (if Hospitalized)" had the final answer option "not hospitalized". However, many respondents did not see that option and left this question blank, although many outpatients did answer this question. Thus, those data should best be looked at as a composite of all patients, rather than distinguishing between in- and outpatients. As a result, we do not have precise data on the breakdown among respondents between outpatient and inpatient, though we can provide a general estimation that at least 80% were outpatient. Finally, the question about types of support was not pilot tested with patients.

## Conclusion

This study finds significant patient interest in a new field, Israeli spiritual care, similar to the level of interest found in countries where the service is well-established, and suggests that increased patient education and awareness will increase that interest. We found that receipt of spiritual care was a positive experience, leaving patients wanting future visits from the spiritual caregiver. As expected, what patients wanted from the spiritual caregiver showed cross-cultural difference, with explicitly religious or spiritual support less frequently desired in Israel. This study helps strengthen the formulation of cross-cultural screening tools, supporting the use of a measure of social isolation and contraindicating the use of demographic or medical data beyond self-identified religiosity/spirituality.

## Competing interests

The authors declare that they have no competing interests.

## Authors’ contributions

MS prepared the initial study design in light of previous studies, distributed the questionnaires, and drafted the manuscript. DLG helped design and coordinate the study. GBS conceived of the study, coordinated its administration, and helped to draft the manuscript. All authors read and approved the final manuscript.

## Authors’ information

Gil Bar-Sela, Clinical Assistant Professor, Deputy Director of the Oncology Section and the Director of the Palliative Care Unit at Rambam Health Care Campus. Michael Schultz, Board Certified Chaplain, Rabbi, M.A., Director of the Spiritual Care Service of the Oncology Section, Rambam Health Care Campus. Doron Lulav-Grinwald, Clinical Psychologist (M.A.), Director of the Psychology Service of the Oncology Section, Rambam Health Care Campus.

## Pre-publication history

The pre-publication history for this paper can be accessed here:

http://www.biomedcentral.com/1472-684X/13/19/prepub

## Supplementary Material

Additional file 1 Patient Questionnaire. Click here for file
