# Supplementary material for: Cultural differences in spiritual care: findings of an Israeli oncologic questionnaire examining patient interest in spiritual care
Source: BMC Palliat Care. 2014 Apr 8;13:19. doi: 10.1186/1472-684X-13-19 (PMC4108186; doi:10.1186/1472-684X-13-19)
Supplement: Additional file 1 — Patient Questionnaire. [file 1472-684X-13-19-S1.docx]

**Annex 1. Patient Questionnaire**

**Patient Questionnaire**

**Integrating Spiritual Care into the Oncology Institute’s Services**

Dear patients,

We are in the midst of adding something new and exciting to the array of services we offer – Spiritual Care – and we would greatly value your help in guiding us as we develop and integrate this service into the oncology center. We have two professionally trained *milavim ruchaniyim* (spiritual care providers) on our palliative care staff who are available to meet with patients and family members. Because *livui ruchani* (spiritual care) is a relatively new field in Israel, and not one which is always well understood, we’ll begin with a short explanation of how we see the things that affect our spirit. The world of the spirit includes our values, beliefs, and attitudes, the sum total of our life experiences, our relationships to the moment, to ourselves, to others, and to nature, as well as our search for and sense of purpose, meaning, and hope in life. For each of us, the sum total of these factors and how they are affecting our spirit could be called an individual's unique current spiritual identity. In situations of crisis or illness, everyone has their own spiritual resources, the things to which they can turn for help in coping. Sometimes it can be hard for us to find the spiritual strength that is to be found within everyone. For some people, spirituality is largely found in religious beliefs and practices, and for others it is found elsewhere. Spiritual care at Rambam aims to meet each individual where they are and support them throughout the period of illness and treatment in a way that respects everyone's values and beliefs. **Our goal is to accompany and support you, on the emotional and spiritual level, throughout this difficult time.**

By answering this questionnaire, you will help us to develop this service in a way that is suited to our unique patient population. In addition, the responses we receive will help to further the research in this field, in Israel and worldwide. You are free to choose whether or not to fill out a questionnaire, and you do not need to identify yourself by name anywhere on the questionnaire.

Thank you for your help!

The Palliative Care Team

**I) Your Spiritual Identity**

1. How would you describe your level of spirituality?

a) Not spiritual b) Somewhat spiritual c) Very spiritual

2. How would you describe your level of religiousness?

a) Secular b) Traditional c) Religious

**II). *Livui Ruchani* (Spiritual Care) and you**

3. There are a number of ways a *milaveh ruchani* (spiritual care provider) can support you. How important are each of these for you? (Circle the appropriate number)

| Role | Not at all | Very little | Somewhat little | In the middle | Somewhat significant | Significant | Very significant |
| --- | --- | --- | --- | --- | --- | --- | --- |
| Listen to your concerns and show care for you | 1 | 2 | 3 | 4 | 5 | 6 | 7 |
| Show care for your family | 1 | 2 | 3 | 4 | 5 | 6 | 7 |
| Help you make difficult decisions | 1 | 2 | 3 | 4 | 5 | 6 | 7 |
| Help you obtain information or help in communicating with staff | 1 | 2 | 3 | 4 | 5 | 6 | 7 |
| Help you reflect on your experience | 1 | 2 | 3 | 4 | 5 | 6 | 7 |
| Help you find meaning in your situation | 1 | 2 | 3 | 4 | 5 | 6 | 7 |
| Help you face your situation with calmness and dignity | 1 | 2 | 3 | 4 | 5 | 6 | 7 |
| Help you find hope or encouragement | 1 | 2 | 3 | 4 | 5 | 6 | 7 |
| Help you cope with your sense of loss | 1 | 2 | 3 | 4 | 5 | 6 | 7 |
| Help you cope with and adjust to the whole situation | 1 | 2 | 3 | 4 | 5 | 6 | 7 |
| Help you find strength to continue | 1 | 2 | 3 | 4 | 5 | 6 | 7 |
| Offer you supportive techniques like relaxation, mediation, music, and guided imagery | 1 | 2 | 3 | 4 | 5 | 6 | 7 |
| Address spiritual or religious questions | 1 | 2 | 3 | 4 | 5 | 6 | 7 |
| Pray with you | 1 | 2 | 3 | 4 | 5 | 6 | 7 |
| To bring a sense of spirituality into the room | 1 | 2 | 3 | 4 | 5 | 6 | 7 |
| Other: ____________ | 1 | 2 | 3 | 4 | 5 | 6 | 7 |

**III) Demographic Information (your confidentiality will be protected)**

4. What is your age? ______

5. Marital Status

a) Single b) Married c) Divorced d) Widow/er

6. How many children do you have (0 if none)? ________

7. Education

a) Primary school b) High school c) More than high school

8. What is your religion?

a) Jewish b) Muslim c) Druze d) Arab Christrian e) Non-Arab Christian f) Other: ________

9. Gender a) Male b) Female

10. Country of birth: _______ If not Israel, at what age did you move to Israel? _______

11. Do you live with another adult (circle one)? Yes No

12. Are you part of a supportive community (circle one)? Yes No

13. Do you have family members living within 25 kilometers of you (circle one)? Yes No

14. If you are hospitalized, how often do you receive visits from family or friends?

a. Almost every day b. A few times a week c. Once a week d. They don't visit e. I'm not hospitalized

15. How much support do you receive from the community, including family and friends?

a. Quite a lot b. A lot c. A medium amount d. Not too much e. Not at all

16. How often do you attend religious services when healthy?

a) Daily b) Weekly c) Once a month d) Once a year d) Never

17. At what stage of your treatment are you?

a) Prechemotherapy b) During chemotherapy c) Receiving radiation therapy d) Receiving chemotherapy and radiation therapy together e) Completed chemotherapy or radiation therapy within the past 6 months f) In the maakav only g) Other: ___________

18. What kind of cancer have you been diagnosed with? __________

19. Is it a recurrence (circle one)? Yes No I don't know

20. Has your cancer metastasiezed (circle one)? Yes No I don't know

21. How worrisome do you think your illness is?

a) Not that worrisome b) Somewhat worrisome but definitely manageable c) Somewhat worrisome, but maybe manageable d) Quite worrisome

22. How long ago were you diagnosed?

a) Within the last 4 weeks b) Within the last 1-3 months c) Within the last 3-6 months d) More than 6 months ago

23. Do you think you have a good understanding of what a *milaveh ruchani* (spiritual care provider) is or does?

a) Not at all b) Not really c) Unsure d) I think so e) Definitely

24. How important do you think it is that the oncology institute include *livui ruchani* (spiritual care) in its services?

a) Not at all b) Not really c) Unsure d) Somewhat important e) Very important

25. How open do you think you would be to a visit from the *milaveh ruchani* (spiritual care provider)?

a) Not at all b) Not really open c) Indifferent d) Maybe interested e) Definitely interested

26. Have you ever had a visit from a spiritual care provider in the past (circle one)?

a) Yes b) No c) I'm not sure
